# Investigation of Multicomponent Fluoridated Borate Glasses through a Design of Mixtures Approach

**DOI:** 10.3390/ma15186247

**Published:** 2022-09-08

**Authors:** Kathleen MacDonald, Daniel Boyd

**Affiliations:** 1 IR Scientific Inc., Halifax, NS B3H 0A8, Canada; 2 Department of Applied Oral Sciences, Dalhousie University, Halifax, NS B3H 4R2, Canada

**Keywords:** borate glass, fluoroborates, bioactive glass, halogen, design of mixtures, ion release, dentistry

## Abstract

Due to their enhanced dissolution, solubility and reaction speed, borate glasses offer potential advantages for the design and development of therapeutic ion-release systems. However, the field remains poorly understood relative to traditional phosphosilicate and silicate bioglasses. The increased structural complexity and relative lack of published data relating to borates, particularly borofluorates, also decreases the accuracy of artificial intelligence models, which are used to predict glass properties. To develop predictive models for borofluorate networks, this paper uses a design of mixtures approach for rapid screening of composition–property relationships, including the development of polynomial equations that comprehensively establish the predictive capabilities for glass transition, density, mass loss and fluoride release. A broad range of glass compositions, extending through the boron anomaly range, were investigated, with the inclusion of 45 to 95 mol% B_2_O_3_ along with 1–50 mol% MgO, CaO and Na_2_O as well as 1–30% KF and NaF. This design space allows for the investigation of the impact of fluorine as well as mixed alkali–alkaline earth effects. Glass formation was found to extend past 30 mol% KF or NaF without a negative impact on glass degradation in contrast to the trends observed in phosphosilicates. The data demonstrates that fluoroborate materials offer an exceptional base for the development of fluoride-releasing materials.

## 1. Introduction

The principal of using therapeutic ion release from bioactive glasses enables the engineering of versatile and tailorable biomaterial platforms to address therapeutic needs at the local tissue level. Such glassy materials react with an aqueous environment to release ionic constituents capable of directing local cellular activity, while concurrently enabling improved processing, sterilization and shelf life relative to traditional drug loading approaches [1,2]. In this regard, antibacterial [3], anti-inflammatory, angiogenic [4] and osteogenic [5,6] materials have all been developed through the inclusion of therapeutic ions into glassy materials [7]; though, to date, this has largely been associated with silicate and phosphosilicate glass chemistries. Increasingly, interest in borate glass chemistries as the basis for controlled release bioactive glasses has become the focus of attention in the literature. 

Commercially, the use of borate glasses (in fiber form) for the management of skin ulcers has proven the utility of borate-based bioactive glasses as therapeutic agents to promote tissue healing and repair [8]. Clearly, the selection of therapeutic ions to be incorporated into a soluble glass network depends on the intended use and indications for use. Two therapeutic inorganic ions of interest for hard tissue engineering in dentistry are magnesium and fluorine. 

Magnesium is known to play a role in the regulation of bone mineral density; both through direct effects on bone cells and indirectly through its impact on bone signaling pathways [9,10]. Along with its ability to alter cell interactions, magnesium ions are capable of substituting into the mineral phase of bone and teeth, forming a magnesium substituted hydroxy apatite and altering the crystal lattice [11,12]. 

Magnesium substitution has been demonstrated to result in smaller crystallite size, imparting both a whiter appearance due to increased light scattering and resulting in an increase in microhardness of enamel [13,14]. Fluoride ions, in turn, are arguably the most well recognized therapeutic ion, with a long history of use in the prevention of tooth decay and have also shown beneficial effects for the maintenance of bone mineral density. For the design of dental materials, optimization of the loading and release of fluoride ions from bioactive glasses has gained both research and commercial interest, both for the prevention of dental decay and acid erosion and for the treatment of dentin hypersensitivity [15,16,17,18,19].

When considering the design of glasses for the release of therapeutic inorganic ions, borate glass networks are an appealing choice due to their high solubility and increased rate of ion release [20]. The substitution of boron oxide into the original bioglass formulation (often referred to as 45S5) in place of the original phosphosilicate glass network for example has been reported to result in a five-fold increase in reaction rate [21]. Furthermore, glasses based on boron networks have been reported to undergo bulk dissolution, proving them a useful base of the design of completely soluble biomaterials [22]. 

The design of borate glasses to meet specific material characteristics, however, is less straightforward than those of their silicate counterparts due to the ability of boron to switch between three- and four-fold coordination [23]. In contrast to the behavior of silicate glasses, the impact of modifier oxide addition to a borate glass impacts glass properties, such as the density, glass transition and network connectivity in a non-monotonic function, with a localized maxima/minima around ~30% modifier addition [24]. 

As the reactivity of bioactive glasses is commonly thought to be attributable to their network connectivity, with the best bioactivity around a network connectivity of two [25], the nonlinear variations in structural changes have a significant impact on glass dissolution and bioactivity. Further complicating the design of borate glasses, the mixed alkali and mixed alkali earth effect results in changes to the location of this minima/maxima when the glass complexity is increased beyond a single modifier binary glass [26,27]. 

As such, while borates are promising materials for the development of ion releasing bioactive glasses, they present an increased challenge for the prediction of composition–structure–property relationships. Difficulties in the prediction of structural drivers of glass reactivity in borate glasses are further complicated when considering magnesium and fluorine-containing borate glasses. Binary magnesium borate glasses have a narrow range of glass formation (45–55% MgO) when formed using traditional melt quench techniques [24,28]. This narrow glass-forming window has been attributed to magnesium’s high field strength and low ionic radius. 

The literature reports are in conflict regarding the role that magnesium plays in a borate glass network, identifying its oxygen bonding as ionic [29] (indicative of a network modifier) and covalent [28] (indicative of network formation). Fluoride addition, in turn, is known to cause the formation of fluoride rich cation domains, removing glass modifiers from the network. 

As the network connectivity of borate glasses varies nonlinearly with oxide modifier addition, it can be expected that the removal of modifying cations into fluoride clustering domains would also impart a nonlinear change in glass structure and properties, making glass behaviors less simple to predict. Some understanding of the impact of fluoride salt addition on the properties of borate glasses can be gained from the field of optical glasses, where fluoroborate glasses have been investigated due to their improved ionic conductivity relative to oxide glasses [30,31,32,33,34]. 

While some structural information can be gained from this work, these studies did not investigate the potential for fluoride release from the materials, or the reactivity of the glass. Furthermore, as there is little consensus on the impact of borate glass structure as a modulating factor for dissolution, extrapolation of composition–structure–property information from existing publications is not feasible. 

For example, investigations on the effect of fluoride salt inclusion on the bioactivity of glasses in a B_2_O_3_–CaO–Na_2_O–CaF–NaF system has been conducted, however the boron content of the glasses was kept constant, limiting our understanding of the impact of the fraction of glass network formers to modifiers [35,36]. The impact of fluoride addition on the degradation, and thus fluoride release, or a borate oxyfluoride glass system is an underexplored research area hindered by our lack of a priori and a posteriori knowledge in the larger field of borate glass networks.

Compounding these issues, borate glasses have historically received little research interest, with less than 2% of published glass science literature from 2007 to 2013 focused on borates [37]. This historical under investigation has left the field at a significant disadvantage in terms of collective knowledge. This disadvantage grows exponentially in the age of machine learning and “materials by design” where the historical knowledge of glass materials can be compiled and interpolated to allow for more refined and rapid identification of useful compositions [38,39]. 

Such a disadvantage is again compounded with the considerations of less studied glass modifiers, such as magnesium and fluoride, where multiple structural roles are possible, increasing the complexity of the structural drivers of material properties. To exemplify this challenge, when using artificial intelligence techniques to model fluoride glass density, Ahmmad et al. found that, while a coefficient of determination (R^2^) of 0.980 was achievable for their dataset of 1258 fluoride glass compositions, it decreased to 0.792 for high-borate glasses [40]. 

A similar study aiming to model the density of oxide glasses found R^2^ values to decrease from 0.84 when modeling binary systems to 0.65 for multicomponent systems (defined as 5 or more components), emphasizing the increased difficulty in predicting multicomponent glass properties [41]. As such, alternative glass design approaches must be used to investigate borate glasses, especially in the context of less-studied glass components, such as fluoride or magnesium.

To better understand the complex relationship(s) governing the composition–property relationships in the understudied field of multicomponent fluoridated borate glasses, a design of mixtures approach is presented in this work. This approach investigates the impact of variations in boron oxide, alkali earth oxide, alkali oxide and fluoride salt loading in a multifactorial design of mixtures methodology. The design of mixtures is a form of experimental design in which all variable components sum to unity (such as the composition of a glass) and can be expressed as relative weights; this feature allows for a mixture of n components to be simplified down to n − 1 variables. 

The compensatory changes required to balance the mixture to unity complicate the use of intuitive, step wise experimental design (such as the replacement of component 1 with component 2, then 3 and then 4). For these reasons, the selection of experimental compositions is typically made though a statistics software program to allow optimal spacing for the generation and assessment of composition-property surface response models. This allows for the development of robust and objectively verifiable composition-property relationships over a broad range of compositions, while minimizing the number of compositions to be studied. 

The compositional range to be investigated covers boron oxide contents of 45–95%, allowing for a broad understanding of the impacts of modifier additions up into the inverted glass range. Minimum inclusion levels of 1% were chosen for all compositions to maintain model focus on multicomponent mixtures (i.e., to prevent the inclusion of binary of ternary compositions), requiring a 95% maximum for B_2_O_3_. 

Oxide modifier ranges were selected between 1 and 50% for oxides magnesium, calcium and sodium, while sodium and potassium fluoride inclusions were limited to 1–30% due to experimental difficulty with high fluoride compositions (unpublished laboratory data) This study aims to serve as an initial screening tool to allow for the identification of regions of interest that merit more in-depth study for their potential use in biomaterials design and development for a variety of applications.

## 2. Materials and Methods

### 2.1. Experimental Design

Composition–property relationships were investigated for borate oxyfluoride glasses in the region of 45–95% B_2_O_3_, 1–50% MgO, 1–50% CaO, 1–50% Na_2_O, 1–30% NaF and 1–30% KF using a design of mixtures approach. Design Expert Software (Stat-Ease, Minneapolis, Minneapolis, MN, USA, Version-12.0.1.0, https://www.statease.com/software/design-expert/ accessed on 6 August 2022) was used to develop an I-optimal quadratic model based on a 6-component mixture, with the design constraints as shown in Table 1. From the model, 31 randomised “runs” corresponding to a glass compositions (Table 2) were identified for investigation.

### 2.2. Glass Synthesis

The 31 glass compositions as specified in Table 2 were made through traditional melt quench methods in the molar ratios shown above. Briefly, appropriate weights of reagent grade modifiers (sodium carbonate, calcium carbonate, magnesium oxide, sodium fluoride and potassium fluoride) were combined with appropriate weights of 98% pure boron oxide (Alfa Aesa), sealed and mixed to homogeneity in HDPE containers using a rotary dry mixer for 1 h (Glas-Col model 099A RD9912). Reagents were then packed in 100 mL 90% Pt/10% Rh crucibles (XRF Scientific) and placed in a Carbolite Gero RHF 14/3 furnace at 600 °C. 

The furnace program was set to dwell for 60 min and then ramp at 20 °C/min to 1200 °C and hold for a further 60 min. Following the final dwelling period, glasses were quenched between two stainless steel plates and stored in desiccated conditions until further processing. Glasses were ground using a two-step process, first using a manual stainless steel glass crusher before further milling with a planetary micro mill using zirconia accessories (Pulverisetter 6 Classic Line, Fritsch, Idar-Oberstein, Germany). The ground glass powder was then sieved with an ASTM E-11 compliant sieve to retain particles of <25 um. All materials were stored in desiccated conditions until the time of further analysis.

### 2.3. X-ray Diffraction

X-ray diffraction was performed using a Bruker D2 diffractometer with a Cu source and Lynx-Eye XE linear array detector. Glass powder samples were analyzed in the region of 10° ≤ 2 θ ≤ 60° with a step width of 0.03 and a step time of 2 s. XRD spectra were analysed using Bruker Diffrac.Eva to assess the relative fraction of crystalline material in the samples (as the ratio of crystalline peaks to amorphous halo in the scan).

### 2.4. Helium Pycnometry

The glass density was measured using an Accupyc 1340 Helium pycnometer (Micromeritics, Norcross, GA, USA) with a 1 cm^3^ sample insert. The pycnometer chamber volume was calibrated with a traceable reference standard as per the manufacturer’s instruction prior to each use. For each glass sample, approximately 0.9 g of ground glass powder was firmly packed into the sample cup and analysed as the average of 10 fill and purge cycles using high purity helium gas (99.995% He, Praxair, Canada).

### 2.5. Differential Scanning Calorimetry

Differential scanning calorimetry was performed using a Pegasus F404 DSC (Netzsch, Selb, Germany), with a silicon carbide furnace in an argon environment. Approximately 25 mg of ground glass was analyzed in a platinum rhodium crucible, fitted with a lid. Glass samples were heated from 50 to 900 °C at a rate of 10 °C/min, with 10 data points collected for each degree (100 data points/min). Thermal curves were assessed to identify the glass transition temperature (as T_onset_, T_inflection_ and T_final_) and first crystallization temperature using the Netzsch Proteus software.

### 2.6. Mass Loss Assessment

The mass loss assessment was performed on all glass compositions in triplicate at timepoints of 1, 12 and 24 h. For each glass sample, 0.1 g of glass powder was placed in a pre weighed 15 mL HDPE centrifuge tube with 10 mL of TRIS-buffered saline solution (BioUltra, Sigma Aldrich, Oakville, ON, Canada), capped and placed horizontally on a shaking incubator at 37 °C and agitated at 2 Hz until the desired time point. 

Following incubation, the samples were centrifuged for 15 min and 300 RCF to remove residual glass from the solution. Tubes were carefully decanted to remove the supernatant, which was capped and stored at 4 °C until the time of fluoride release analysis. Test tubes with glass residuals were dried in an oven at 70 °C until constant weight was achieved (approximately 2 weeks) to determine residual glass mass and calculate the % of weight loss.

### 2.7. Fluoride Release

The fluoride release was assessed using the decanted supernatants from the mass loss assessment. The concentration of F released into solution was measured using an Accumet AB250 pH/ion selective electrode meter equipped with a fluoride-ion-selective electrode (Accumet AB250). The ion-selective electrode was calibrated using a series of NaF standard solutions ranging from 0.1 to 10,000 ppm (NaF 0.1 M F, Sigma Aldrich, Oakville, ON, Canada). All samples were mixed with TISAB III as per the manufacturer’s instruction prior to analysis.

## 3. Results

### 3.1. Glass Synthesis

All compositions were successfully melted, with the exception of composition #20, which did not form a liquid melt at 1200 °C. Composition 20 was excluded from further analysis, and no attempt to remelt the composition at a higher temperature was made due to the potential impacts of variable thermal processing on glass properties. Multiple compositions did not form homogeneous clear glasses, evidenced both through nonuniform and cloudy appearance (denoted by an asterisk next to composition ID in Table 3).

### 3.2. XRD Analysis

**Figure 1:**
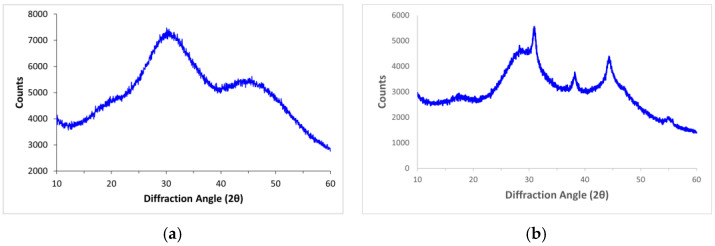
Representative XRD spectrum collected from ground powder from quenched glass of (**a**) composition 9 and (**b**) composition 10.

XRD analysis of the 30 successfully melted oxide blends revealed varying degrees of crystallinity, ranging from 1.4 to 88% (Figure 1, Table 3). A reduced quadratic model was developed to understand the influence of glass components on melt crystallization, as shown in Table 4. While the R^2^ of 0.88 suggests a strong correlation between the modeled and true crystallization values, a large difference between the adjusted R^2^ and predicted R^2^ suggests an impact of outliers on the model, identified as composition 1. 

**Figure 2:**
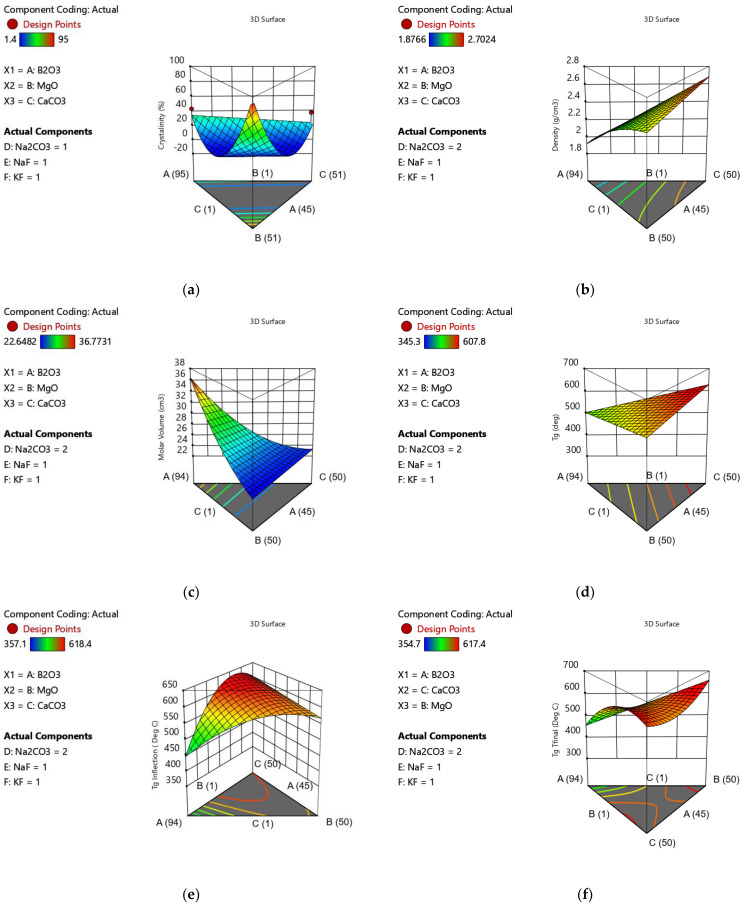
3D surface plots demonstrating the nonlinear relationships between the glass composition and (**a**) crystallinity, (**b**) density, (**c**) molar volume, (**d**) Tg onset, (**e**) Tf inflection and (**f**) Tg final.

The removal of outliers did not result in an improvement in the model’s predictive ability; however, the adequate precision result of 20.10 suggests that the model is useful in providing an estimate of the crystallinity of materials in this composition space. The factors that had a positive effect on crystallinity (favoured crystallization) in ranking of degree of impact were MgO > B_2_O_3_ > CaO > KF, factors that had a negative effect on crystallinity (favoured amorphous glass formation) in ranking of degree of impact were Na_2_O > NaF > B_2_O_3_ × MgO > MgO × KF > MgO × CaO > MgO × NaF > MgO × Na_2_O, as represented in the 3D surface plot of Figure 2a. 

**Figure 3:**
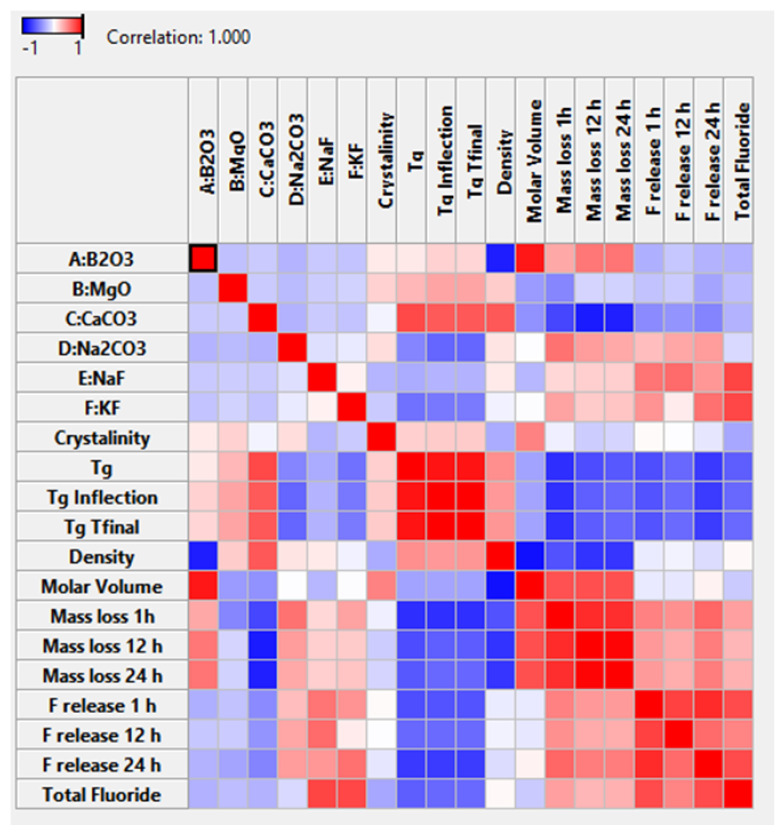
Correlation table visualising the impact of glass components on the measured glass properties with correlations displayed from blue for negative correlations to red for positive correlations.

To better visualise the impact of single glass components with opposing impacts as combination factors a graphical correlation table is presented in Figure 2. For example, it can be visualised that the impact of KF additions imparts a positive linear effect, along with a negative second order effect for MgO × KF, resulting in an overall negative correlation to crystallinity (sixth row, seventh column of Figure 3).

### 3.3. Helium Pycnometry

The densities measured ranged from 1.88 to 2.70 g/cm^3^ as shown in Table 3 (with the exceptions of 1, 6 and 13, which yielded insufficient material for analysis). The density data was fitted to a reduced quadratic model as presented in Table 4. All glass component terms had a positive effect on glass density, with the effects ranked from most to least significant as CaO > NaF > KF > MgO > Na_2_O > B_2_O_3_ > B_2_O_3_ × Na_2_O > B_2_O_3_ × CaO. The only equation term with a negative impact on the glass density was CaO × Na_2_O, as visualised in Figure 1b. The molar volume ranged from 22.64 cm^3^/mol to 36.77 cm^3^/mol, as shown in Table 3. 

The molar volume was fitted to a quadratic model as presented in Table 4. All individual glass components had a positive effect on molar volume, along with the Na_2_O × CaO interaction term. The relative magnitude of the terms positively impacting molar volume was B_2_O_3_ > CaO > Na_2_O > KF > MgO > NaF > CaO × Na_2_O. The terms that had negative effects on molar volume (caused a contraction of the glass network) were all interaction terms, their ranking from greatest negative impact to least impact is B_2_O_3_ × Ca_2_O > B_2_O_3_ × MgO > B_2_O_3_ × Na_2_O > B_2_O_3_ × NaF > B_2_O_3_ × KF, as visualised in Figure 2c. To better visualise overall impact of glass components, a graphical correlation table is presented in Figure 3.

### 3.4. Differential Scanning Calorimetry

**Figure 4:**
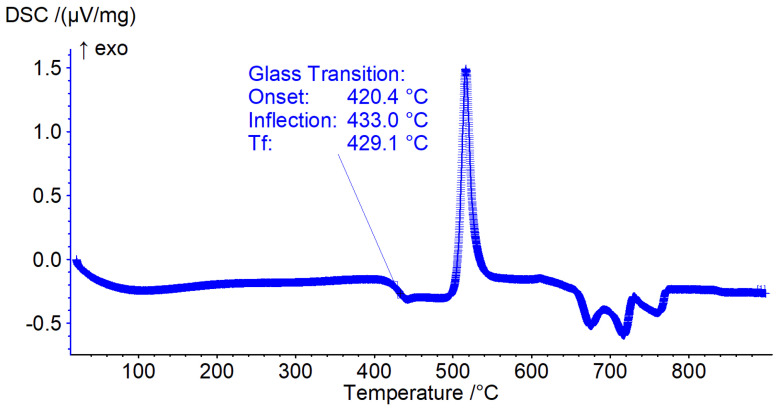
The representative DSC trace collected from the powder of the quenched glass of composition 9.

The Tg onset values for the glasses investigated ranged from 346.3 to 607.8 °C (Figure 4, Table 3) (exceptions of compositions 1, 13, 17 and 20, which yielded insufficient material for processing and analysis) and was fitted to a linear model as shown in Table 4. All glass components had a positive effect on the Tg onset (increased Tg) with the effects ranked from most to least significant as CaO > MgO > B_2_O_3_ > NaF > Na_2_O > KF as visualised in Figure 2d. Tg inflection ranged from 357.1 to 618.4 °C (Table 3) and was best fitted to a reduced quadratic model. The glass components with a positive effect on Tg inflection, ranked from greatest to least impact were B_2_O_3_ > NaF > MgO > KF > CaO > B_2_O_3_ × CaO > B_2_O_3_ × Na_2_O > CaO × NaF > B_2_O_3_ × MgO > MgO × KF, factors with a negative impact on Tg inflection ranked from greatest to least impact were Na_2_O > CaO × Na_2_O > MgO × Na_2_O, as visualised in Figure 2e. 

The glass transition as measured by Tg final ranged from 354.7 to 617.4 °C and was best fitted by a reduced quadratic equation. The factors that favoured an increase in Tg final, in ranked form greatest to least impact were MgO > B_2_O_3_ > NaF > KF > CaO > B_2_O_3_ × CaO > B_2_O_3_ × Na_2_O> CaO × NaF. The factors that favoured a decrease in Tg final, in order of greatest to least impact were Na_2_O > MgO × Na_2_O > CaO × Na_2_O > MgO × NaF > MgO × CaO as visualised in Figure 2f. To better visualise the overall impact of the glass components, a graphical correlation table is presented in Figure 3.

### 3.5. Mass Loss Assessment

The mass loss assessment was performed on all glasses with the exceptions of 1, 6, 9, 13, 17 and 20 due to insufficient material yield for processing and analysis. The mass loss was from 40.5% to 99.8%, 55% to 99.5% and 59% to 99.4% following 1, 12 and 24 h, respectively (Table 5). The mass loss for all timepoints was best modelled following a logit transformation with lower and upper limits of 0 and 100% (Table 4). The mass loss following 1 h was best fitted to a reduced quadratic model, and the factors favouring increased mass loss ranked from greatest to least impact were CaO > MgO > B_2_O_3_ > Na_2_O. 

**Figure 5:**
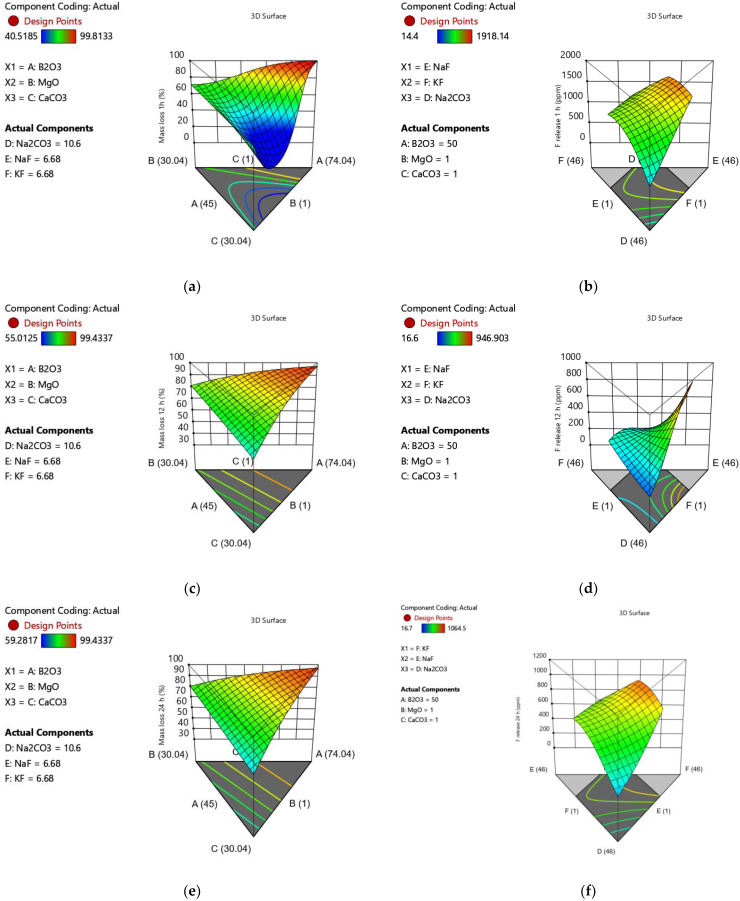
3D surface plots demonstrating the nonlinear relationships between the glass composition and (**a**) 1 h mass loss, (**b**) 1 h fluoride release, (**c**) 12 h mass loss, (**d**) 12 h fluoride release, (**e**) 24 h mass loss and (**f**) 24 h fluoride release.

The factors that favoured a decrease in mass loss were NaF > KF > B_2_O_3_ × CaO > B_2_O_3_ × MgO > CaO × Na_2_O as visualised in Figure 5a. The mass loss following 12 h was best fitted to a linear model, with the factor favouring increased mass loss ranked from greatest to least impact being Na_2_O > B_2_O_3_ > NaF. The factors that resulted in a decrease in mass loss were CaO > MgO > KF as visualised in Figure 5c. The mass loss at 24 h was best modeled using a reduced quadratic model, factors that had a positive impact on, ranked from greatest to least impact were B_2_O_3_ > Na_2_O > NaF > KF, and the factors that favoured a decrease in mass loss, ranked from greatest to least impact were CaO > MgO as visualised in Figure 5e.

### 3.6. Fluoride Release

The fluoride release was assessed for all glasses with the exceptions of 1, 6, 9, 13 and 20 due to insufficient materials for analysis. The fluoride release was measured at timepoints of 1, 12 and 24 h and ranged from 14.4 to 1918.14 ppm at one hour, 16.6 to 946 ppm at 12 h and 16.7 to 1064.5 ppm after 24 h (Table 5). The fluoride release was best modelled following a logit transformation with upper and lower bounds of 0 and 2000 ppm (Table 4). The glass components favouring an increase in fluoride release at 1 h, in order of greatest to least impactful were Na_2_O > NaF > KF> Na_2_O × KF, components favouring a decrease in fluoride release were B_2_O_3_ > CaO > MgO > CaO × NaF as visualised in Figure 5b. 

The fluoride release following 12 h of incubation in TRIS-buffered saline was fitted to a similar reduced quadratic model following a logit transformation with upper and lower bounds of 0 and 2000 ppm. The factors that favoured an increased in F release following 12 h in order of greatest to least impact were Na_2_O > KF > B_2_O_3_ × Na_2_O > MgO × Na_2_O > Na_2_O × NaF, factors that negatively impacted fluoride release at 12 h were, in order of greatest to least impact NaF > B_2_O_3_ > CaO > MgO > NaF × KF > B_2_O_3_ × Na_2_O as visualised in Figure 5d. 

The fluoride release following 24 h of incubation in TRIS-buffered saline was fitted to a similar reduced quadratic model following a logit transformation with upper and lower bounds of 0 and 2000 ppm. The factors that favoured an increased in F release following 12 h in order of greatest to least impact were KF > Na_2_O > B_2_O_3_ × NaF > Na_2_O × NaF, and the factors that favoured a decrease in F release at 24 h, from greatest to least impact were NaF > B_2_O_3_ > CaO > MgO > CaO × KF > MgO × KF as visualised in Figure 5f. To better visualise overall impact of glass components, a graphical correlation table is presented in Figure 3.

## 4. Discussion

The prediction of bioactive glass reactivity is frequently correlated to the network connectivity in phosphosilicate glasses. Stemming from Hill’s alternative view on bioactive glass dissolution, it is proposed that a network connectivity around 2 allows the glass to behave as a tangled linear polymer, allowing for easy release of glass modifiers and the formation of the silica gel layer as a precursor to calcium phosphate deposition [25]. Similar correlations between borate glass network structure and glass dissolution have been found, suggesting that network connectivity is a strong predictor of the rate of glass dissolution in borate systems as well [22]. 

As such, structural modifications are a natural design consideration for the development of novel bioactive glasses. Additions of modifying oxides, provide for a linear decrease in network connectivity, which can be empirically calculated in silicate glasses, providing strong predictors of glass properties. The addition of fluoride to the network of a phosphosilicate has the opposing effect, removing network modifiers from the glass structure and increasing network connectivity, again with a linear effect. For borate glasses, direct manipulation of the glass network structure through modifier additions is less straightforward than in silicate glasses. 

In borate glasses, network modifiers may act to generate a transformation from three- to four-fold coordination of boron, or to generate non-bridging oxygens. The balance between these two structural roles is dependent on the modifier and the amount added to the formulation chemistry. While some rules governing the formation of tetrahedrally-coordinated boron and non-bridging oxygen have been generated, they do not offer a consensus, and they cannot be extrapolated across all borate glasses [42]. 

For example, while many these models treat glass modifiers equally, it is recognized that the fraction of tetrahedrally-coordinated boron is dependent also on the modifier chosen, as demonstrated by the comparison of binary lithium and caesium borate glasses, where the lithium glasses showed strong agreement to models previously developed, but the caesium glasses had substantially fewer tetrahedrally-coordinated boron (46% vs. 43% tetrahedrally-coordinated boron at 41% modifier addition for lithium and caesium borates, respectively) [43]. 

Following from this, the effects of fluoride additions cannot be easily predicted in borate glasses. If, as suggested in some literature, fluoride ions serve to remove cationic modifiers from the glass network, they can serve both to decrease the number of non-bridging oxygens or to decrease the number of tetrahedral coordinated boron, having opposing effects on the overall network connectivity of the glass. For this reason, the selection of a region of interest for investigation in a borate glass network is much less easily identified than their silicate counter parts (where centering the network connectivity around 2 would be a reasonable guess to begin investigation). 

In magnesium oxyfluoride borate glasses, yet another level of structural complexity is added, which further obscures predictions of composition-structure property relationships. Magnesium oxide within a borate glass network has been shown to act both as a glass modifier [29] (decreasing network connectivity) and a network former [28] (increasing network connectivity). Furthermore, when forming cation-anion clusters with fluoride, magnesium may exist in a four- or six-fold coordinated state [44]. 

Due to the multiple confounding structural variables in these systems, more structured and guided investigations for the identification of useful compositions is needed. While artificial intelligence models have been used for this purpose in silicate glasses, allowing from the predictions of glass properties across broad ranges of glass compositions, they have a lower modeling accuracy for more complex networks, such as multicomponent, fluoridated and borate glasses. As such, rapid screening across a broad range of compositions through design of mixtures experiments remains a relevant method of maximizing research efficacy, allowing for the selection of specific regions of interest for further investigation.

Most materials studied in this work were predominantly amorphous, with limited crystalline phases. Limits of inclusion to minimize crystallinity (defined in this case as below the 3% limit of accuracy of the methods used) were successfully identified using the polynomial equations in Table 4 and found to be: 82% for B_2_O_3_, 27% for MgO, 41% for CaO, 43% for Na_2_O. Upper limits for NaF and KF inclusion in this system were not identifiable (i.e., above 30% maximum investigated). The crystallinity models demonstrated a decreased tendency to crystalize with increased fluoride addition. 

These findings counter previous investigations into the effect of fluoride addition in borate glasses. Reports of the effects of CaF_2_ addition on a SrO-B_2_O_3_ glass ceramics at either 2 or 5 mol% was also found to increase nucleation, improving the ease of formation of nanoceramics [45]. Glass forming ability also became increasingly difficult as fluoride was added when investigating the effect of fluoride addition to lithium borate glasses [46]. Such reports are consistent in the literature for bioactive glasses formed from phosphate and silicate networks. 

Phase separation and crystallization was reported when CaF_2_ content was increased beyond 10 mol% in a phosphate glass [47]. Glass formation has also been reported to be hindered by CaF_2_ inclusion beyond 24% in phosphosilicate bioactive glasses [48,49], however it has been suggested that improper glass network design is responsible due to the increase in network connectivity observed when replacing CaO with CaF_2_ [49]. Some synthesis techniques allow for high rates of fluoride inclusion in borate glasses; notably, heavy metal borate glasses have been produced with up to 70% fluoride salts, however large cation radii of the heavy metal have been proposed to stabilize fluoride inclusion [30,31,32,33].

The impact of glass modifier oxides on glass density increased with increasing molar mass, suggesting that their impact is in part a result of atomic mass, as would be expected. This trend was not followed by NaF and KF, which imparted greater increases in density than would be expected from their atomic mass alone. Models developed for molar volume in this study found that B_2_O_3_ had the greatest positive effect on molar volume, that is to say, all glass modifiers investigated resulted in decreased molar volume relative to B_2_O_3_. 

This finding is consistent with knowledge on borate glass structure, where glass modifier addition is known to increase boron coordination, increasing the packing efficiency. It is noteworthy that this trend is continuous in this glass series, with no reversal when studying borate contents from 95 to 45%, without evidence of a boron anomaly. While further structural investigation is required to better understand this finding, it appears that tetrahedral borate coordination continues to be the main function of modifier oxide addition through the glass series.

While cation radius has previously been used predict molar density in borate glass networks [50,51], no such trend was observed in this study. When compared to oxide glasses, molar volumes for fluoroborate glasses show less consistent trends. El-Egili et al. found an initial decrease of molar volume with increasing BaF_2_ a binary borate glass, but found this trend to reverse after a local minima at 40% BaF_2_, a finding attributed to the formation of BaF_4_ tetrahedra [31]. 

In further investigation of tertiary borate glasses, the substitution of PbF_2_ for CdF_2_ or BaF_2_ for PbF_2_ increased molar volume, a finding attributed to the larger size of the structural units formed [30,33]. The strong impact of NaF and KF on density may be explained by their impact on the molar volume. When looking at the relative effects of Na_2_O and NaF on molar volume, their coefficients in the predictive model are 0.31 and 0.18, respectively, indicating the NaF addition results in significantly smaller molar volume than Na_2_O. 

The exact nature for this decrease in molar volume with NaF addition however is unclear. If fluoride ions segregate from the glass network into cation clusters, the addition of fluoride salts would be expected to decrease the fraction of tetrahedrally-coordinated boron, which would cause the glass network to expand. Alternatively, fluoride addition may favor the removal of non-bridging oxygen from the network, however the linear trend in molar volume and boron content suggests that tetrahedrally-coordinated boron’s likely dominate the structure.

Previous work suggests that molar volume may be predicted from the unit volumes of structural units formed within the glass, which can be garnered from binary borate glasses formed of each of their components [30,31,32,33,34,52]. This modeling however requires accurate structural knowledge of the glass, which is difficult to obtain for multicomponent borate glasses. Machine learning algorithms have been applied to predict glass density from large data sets, on both oxide glasses and fluoridated glasses [40,41,53]. While these studies have been relatively successful, the algorithms developed are skewed by the data sets on which they are trained. 

They therefore can present accurate predictions for glasses that fall within the pallet of previously studied glasses but may (and do) fail to accurately model glasses with more novel compositions. Such an issue is highlighted in the works by Ahmmad et al. who used artificial intelligence techniques to predict the density of fluoride glasses from their chemical composition and ionic radii, but found that modeling of borate rich glasses was significantly less accurate [40]. 

A similar decrease in model accuracy was observed when attempting to predict the densities of multicomponent glasses when compared to binary, ternary, or quaternary materials [41]. Two factors may be contributing to the failure of the predictive models in both of these cases, (1) a relative lack of data for model fitting in borate and multicomponent glasses and (2) the added structural complexity of the glasses themselves, including the “boron anomaly” and mixed alkali/alkali earth effects. 

Artificial intelligence algorithms are complex mathematic models that find patterns in the data on which they are “trained”, which when developed appropriately can be used to predict outcomes (in this case glass density) from inputs (in this case glass compositions). As such the failing of this approach is not surprising in the field of borates, which have been described as “enigmatic materials”, where discussion of structural changes center on the “borate anomaly” [24]. 

Similar difficulties can be expected with the prediction of magnesium containing glasses, where controversy lies in its bonding coordination and trends followed by other alkali earth oxides are broken [28,29,54]. Magnesium oxide, unlike oxides of Ca, Sr and Ba, is often identified as an intermediate glass modifier, capable of both tetrahedral coordination with oxygen as a glass network former and octahedral coordination with oxygen as a network modifier, further impacting glass network density [55]. 

As such, while the models are helpful in providing a prediction in density trends, their accuracy decreases for more unusual or novel glasses. Simply put, if artificial intelligence models are designed to predict expected results and can not be relied on to identify unusual behaviors. As such, the models presented in this work, offer a unique insight to navigate multicomponent borate glasses and allow for the identification of regions of interest for further investigation.

Glass transitions onset ranged from 346 to 607 °C. These temperatures are considerably lower than glass transition temperatures observed for binary borate glasses made from alkali and alkali earth metal oxides, which range from approximately 560 to 925 °C [28]. Alkali earth oxides had the greatest impact on glass transition, which could be explained by the formation of strongly bonded oxygen centers, increasing the network stiffness [26,27]. 

Similarly, low glass transition temperatures ranging from 382 to 660 °C were reported by Manchester et al. in the investigation of mixed alkali-alkali earth oxide effects in a K_2_O-SrO-B_2_O_3_ system, attributed to the lower fraction of B4 coordinated boron caused by the mixed alkali-alkali earth oxide effect [26]. This effect, thought to be of a similar nature to the mixed alkali effect, likely occurs due to the increased stability of adjacent B3 coordinated boron modified by the polarization effect caused by dissimilar metal ion modifiers [56]. 

In borate glasses, glass transition temperature is recognized to be closely linked to the fraction of B4 in the glass network, which increase rigidity. As such, glass transition temperature varies nonlinearly with modifier oxide addition similarly to the formation of B4 tetrahedra, reaching a maximum at approximately 30% oxide addition. Magnesium oxide is unique in that the glass-forming window is limited to 45–55% MgO, and the maximum B4 fraction is observed at ~50% MgO [42]. 

While rapid cooling techniques have been used to expand the glass forming region, they have resulted in significant decreases in glass transition (approximately 600 °C in the region of 50% and below 300 °C in the region of 0–30% MgO addition) [51]. The low glass transition temperatures observed in this study may be related to the low glass transition temperatures observed following low-level MgO inclusion in binary glasses, however the overall impact of magnesium addition in this work suggests it serves to increase the Tg. 

This positive correlation (as visualised in Figure 1 and Figure 2) could be explained by the formation of covalent bonds as a network former, as well as by the stiffening effect of divalent cation bridges, as observed with calcium addition. While these finding suggest that magnesium in likely to act as a glass modifier in a multicomponent borate glass, such as the one studied (as its impact on glass transition is similar in magnitude and direction to that of calcium—a known network modifier), further structural analysis is required to fully understand the interaction between the simultaneous inclusion of magnesium among other glass components.

Fluoride addition offers another alternative explanation for the decreased glass transitions observed in this study. A decrease in Tg with increasing fluoride content was also observed in investigation of fluoridated lithium borate glasses [46]. Similar decreases in Tg with the addition of BaF_2_ in a binary glass has been attributed to the formation of terminal B-F bonds at the expense of B-O bonds [31]. Similar trends are found in silicate and phosphosilicate glasses [49,57], where it is attributed to the binding of fluorine ions to calcium in the glass network, preventing the calcium from acting as an ionic bridge between two adjacent non bridging oxygens and thus decreasing network rigidity [49]. 

A study on the impact of alkali earth fluorides (MgF_2_, CaF_2_, SrF_2_, BaF_2_) on the electrical properties of the MeF_2_-Na_2_B_4_O_7_ system similarly suggests that fluoride occupies some direct boron bonding sites, in the form of Na+[F-BO_3/2_] [44]. The MgF_2_ containing glasses were noted to retain more fluoride than their Ca, Sr or Ba counterparts during glass melting, due to the formation of octahedrally coordinated MgF_6/2_ centers [44]. Magnesium fluoride centers may decrease the impact of magnesium addition on the fraction of B4 however further investigation would be required to demonstrate if the additions of NaF or KF along with MgO has the same potential of forming MgF_6/2_.

Direct comparison of mass loss between the compounds studied and previously materials reported in the literature is difficult to achieve due to the lack of standardization in test methods, including the selection of elution media, particle size and mass (or surface) to volume ratios. Effort to standardize these variables using ISO 10993-14 has provided beneficial insight, however the methods are developed for more resilient glass ceramic systems and are not designed to study rapidly degrading materials. 

With that said, some general trends may be compared to gain insight into the changes observed in these glasses. The glasses in this study demonstrated fast and extensive mass loss ranging from 41% to 100% after a single hour and rising to 60–100% following 24 h. This rapid dissolution could be beneficial for the use of glasses as ion delivery vehicles in oral care application, where daily exposure to the glasses could present issues related to the accumulation of unreacted glass particles [58]. 

While the high reactivity of borate glasses with low modifier inclusion is not unexpected, the ability of this glass system to create glasses of high calcium and fluoride inclusions while maintaining a high degree of reactivity is promising. While binary magnesium borate glasses show unusually high rates of dissolution, calcium oxide has been reported to have the greatest impact on supressing glass dissolution in alkali and alkali earth oxide borates [22]. This effect was similarly observed with the addition of calcium in this work, however extensive dissolution was maintained, providing a platform for controlled dissolution in a relatively high calcium content glass.

The strongest predictor of high rates of mass loss was associated with the B content in the glass, which is consistent with previous reports of boron being a soluble component of the glass [20]. Similarly, Na_2_O, NaF and KF served to increase mass loss when added to the glass network. Notably, KF increased mass loss at early time points, whereas B_2_O_3_ has a greater effect at extended time points. The finding of increased glass degradation with the addition of fluoride salts counters the findings in the study of phosphosilicate glasses, where fluoride addition (typically in the form of CaF_2_) decreases the rate of silica release into solution [59,60]. 

Two factors have been identified in the literature to explain these finding: changes to the glass network structure and changes to the pH of the extraction solution. Increases in network connectivity have been proposed to explain the decrease in reactivity in glass series where CaF_2_ is added into the glass at the expense of CaO or Na_2_O [61]. Similar findings are also observed in studies where the network connectivity is kept constant, suggesting this may be only partially responsible for the observations [59,60]. The decrease in reactivity for glasses in which the network connectivity is not altered may be explained by the ability of F– ions to exchange with OH– ions in solution, thereby decreasing the pH of the solution and causing the formation of a protective silica gel layer [59]. 

In contrast with phosphosilicate glasses, borate glass dissolution is not limited by the formation on a pacifying gel layer and results in a lesser increase in solution pH during dissolution. As such, borate glass networks can allow for a greater addition of fluoride to the glass network without compromising reactivity. This is a desirable feature of the design of a fluoride-releasing material, as full dissolution allows for greater release of fluoride into a bioavailable state (i.e., not retained in unreacted glass).

When investigating the weight loss in a fluoridated borate glass based off of full substitution of B_2_O_3_ for SiO_2_ in the original 45S5 composition with 2 wt% of fluoride salt weight loss was reported to be in the order of LiF > NaF > CaF_2_ > ZnF_2_, however no fluoride free composition was provided for comparison [45]. It is notable that due to the choice to use an equal weight percentage for the addition of fluoride salts, the amount of fluoride incorporated into the system would follow the same trend. 

**Figure 6:**
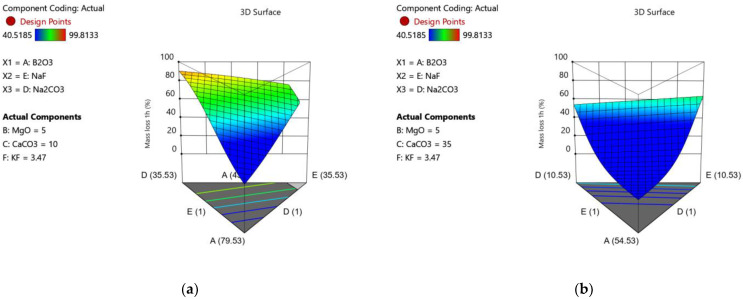
Visualization of the effects of Na_2_O and NF inclusion on mass loss at 1 h at (**a**) low and (**b**) high CaO loadings.

To best visualize the effects of fluoride addition on mass loss in their work, the 3D surface responses at both high and low calcium concentration can be observed as in Figure 6. At low calcium concentration (exemplified as 10% CaO), Na_2_O glasses demonstrate greater mass loss than their NaF counterparts. At high calcium concentration, (exemplified as 35% CaO), NaF glasses demonstrate greater mass loss than their Na_2_O counterparts. 

This finding is counter intuitive to finding that glass dissolution is dependent on the solubilities of the dissolution products in solution, where the combination of Ca and F would be expected to decrease the solubility of the glass. In turn, structural effect, namely the decrease in the B:O ratio, and a decrease in the fraction of B4 coordinated fraction is likely to contribute to the change in dissolution. For a better understanding of the underlying mechanism through which fluoride addition alters glass dissolution structural analysis of the glasses is needed.

## 5. Conclusions

A design of mixtures approach allowed for the development of strong predictive models to assess the composition–property relationships in multicomponent fluoroborate glasses. Complex nonlinear relationships demonstrated the increased complexity in the borate glass systems relative to their silicate glass counterparts. High rates of fluoride inclusion, without negatively impacting glass formation or mass loss, demonstrated that mixed alkali–alkaline earth fluoroborate glasses offer an exceptional base for the design of fluoride-releasing glasses.

## Figures and Tables

**Table 1:** Design of the mixture components.

Component	Minimum (mol%)	Maximum (mol%)	Coded Low	Coded High	Mean	StdDev.
B_2_O_3_	45	95	+0 ↔ 45	+1 ↔ 95	54.14	13.12
MgO	1	50	+0 ↔ 1	+0.98 ↔ 50	9.33	12.33
CaCO_3_	1	50	+0 ↔ 1	+0.98 ↔ 50	9.77	12.66
Na_2_CO_3_	1	50	+0 ↔ 1	+0.98 ↔ 50	11.18	12.71
NaF	1	30	+0 ↔ 1	+0.58 ↔ 30	7.95	8.80
KF	1	30	+0 ↔ 1	+0.58 ↔ 30	7.63	8.64
	Total = 100%		L Pseudo Coding			

**Table 2:** Compositions of the 31 investigational glasses, denoted in molar % as identified by the design of mixtures outlined in Table 1 (repeated compositions demoted by matching Greek letters).

	B_2_O_3_	MgO	CaCO_3_	Na_2_CO_3_	NaF	KF
1	46.0000	1.00000	1.00000	50.00000	1.00000	1.00000
2	67.88520	1.32178	1.82480	23.48440	1.00000	4.48374
3	54.24860	11.60190	9.07179	2.35758	21.72010	1.00000
4	45.00000	5.30483	1.00000	17.69520	30.00000	1.00000
5 ^α^	45.00000	23.09420	1.00000	22.93810	3.04775	4.91997
6 ^β^	68.64670	1.00000	23.11950	1.00000	2.95458	3.27929
7	72.52560	1.00000	1.00000	6.88452	17.58990	1.00000
8	48.20090	4.00991	13.57280	9.38025	11.04280	13.79340
9 ^α^	45.00000	23.09420	1.00000	22.93810	3.04775	4.91997
10	45.00000	3.99303	5.03366	1.02125	14.95210	30.00000
11	45.00000	1.00000	1.00000	22.00000	1.00000	30.00000
12	52.19040	1.00000	1.00000	1.00000	30.0000	14.80960
13 ^β^	68.64670	1.00000	23.11950	1.00000	2.95458	3.27929
14 ^γ^	45.88610	23.51110	24.03280	2.00722	3.56274	1.00000
15	45.00000	27.52700	1.00000	1.00000	18.73830	6.73472
16 ^δ^	45.00000	1.00000	23.61650	23.34480	4.33026	2.70839
17	95.00000	1.00000	1.00000	1.00000	1.00000	1.00000
18	46.24920	1.00000	26.88010	1.00000	20.22320	4.64742
19 ^ε^	45.00000	1.00000	1.00000	25.29430	14.07570	13.63000
20	45.00000	50.00000	1.00000	2.00000	1.00000	1.00000
21	45.00000	4.03483	29.27670	2.65946	1.00000	18.02900
22	72.34390	1.00000	1.00000	1.00000	4.83657	19.81960
23	54.17490	1.00000	3.36218	33.29040	7.17249	1.00000
24 ^ε^	45.00000	1.00000	1.00000	25.29430	14.07570	13.63000
25	46.00000	1.00000	50.00000	1.00000	1.00000	1.00000
26	48.47710	28.60270	2.03278	1.00000	1.00000	18.88740
27	59.11740	13.30200	1.00000	11.26980	1.00000	14.31080
28	68.32760	23.23240	1.18548	2.06453	4.18996	1.00000
29	78.38850	7.96152	5.19700	6.45299	1.00000	1.00000
30 ^γ^	45.88610	23.51110	24.03280	2.00722	3.56274	1.00000
31 ^δ^	45.00000	1.00000	23.61650	23.34480	4.33026	2.70839

**Table 3:** The material characterization results.

	% Crystallinity	Density (g/cm^3^)	Molar Volume (cm^3^)	Tg Onset (°C)	Tg Inflection (°C)	Tg Final (°C)
1 *	88.0	-	-	-	-	-
2	9.0	2.26	29.45	472.4	489.4	479.9
3	2.0	2.40	24.49	505.7	521.5	513.0
4	3.0	2.45	23.77	392.4	404.7	403.1
5	7.9	2.42	24.66	422.9	434.4	431.6
6 *	28.0	-	-	589.2	604.9	599.4
7 *	25.3	2.18	29.26	433.1	453.1	444.9
8 *	17.9	2.50	24.61	429.1	444.1	435.2
9	3.5	2.44	24.41	420.4	433.0	429.1
10 *	19.0	2.41	25.02	381.3	400.6	394.4
11 *	13.1	2.36	27.08	345.3	357.1	354.7
12	2.3	2.30	25.77	384.2	399.4	395.6
13 *	19.9	-	-	-	-	-
14	2.0	2.57	22.68	566.9	583.5	575.6
15	5.0	2.45	22.65	480.9	494.9	490.4
16	4.9	2.46	25.56	430.2	441.0	440.3
17 *	42.9	1.88	36.77	-	-	-
18	5.0	2.50	23.90	555.1	569.8	566.5
19	1.9	2.40	25.85	358.9	369.9	367.1
20	-	-	-	-	-	-
21	1.9	2.51	24.75	489.8	503.2	501.9
22 *	18.4	2.12	30.95	387.5	400.2	400.6
23	3.6	2.41	26.77	419.4	433.1	427.0
24	1.4	2.42	25.59	357.7	369.9	367.8
25 *	38.1	2.70	23.03	607.8	618.4	617.4
26	4.2	2.34	25.02	509.1	523.8	521.9
27	1.7	2.32	27.11	457.6	471.7	468.1
28	2.4	2.27	27.01	538.8	558.8	556.4
29	2.5	2.17	30.32	476.0	501.5	492.1
30	2.3	2.57	22.70	568.6	582.4	576.7
31	7.1	2.46	25.64	582.4	439.8	435.2

* Compositions found to be cloudy or coloured upon quenching. Note: Missing values are not reported for compositions that yielded insufficient material for analysis.

**Table 4:** Regression model equations (actual equations) generated through design expert for the prediction of glass properties, along with the model fit statistics.

Response	Regression Model	R^2^	R^2^ Adjusted	R^2^ Predicted	Adequate Precision
Crystallinity	0.40 × B_2_O_3_ + 6.42 × MgO + 0.19 × CaCO_3_ − 0.45 × Na_2_CO_3_ − 0.27 × NaF + 0.12 × KF − 0.10 × B_2_O_3_ × MgO − 0.09 × MgO × CaCO_3_ − 0.05 × MgO × Na_2_CO_3_ − 0.06 × MgO × NaF − 0.10 × MgO × KF	0.88	0.86	0.44	20.10
Density	0.018 × B_2_O_3_ + 0.023 × MgO + 0.035 × CaCO_3_ + 0.021 × Na_2_CO_3_ + 0.029 × NaF + 0.027 × KF + 0.00015 × B_2_O_3_ × MgO + 0.00018 × B_2_O_3_ × Na_2_CO_3_ − 0.00017 × CaCO_3_ × Na_2_CO_3_	0.98	0.97	0.95	45.83
Molar Volume	0.39 × B_2_O_3_ + 0.23 × MgO + 0.34 × CaCO_3_ + 0.31 × Na_2_CO_3_ + 0.18 × NaF + 0.26 × KF − 0.0033 × B_2_O_3_ × MgO + -0.0054 × B_2_O_3_ × CaCO_3_ − 0.0031 × B_2_O_3_ × Na_2_CO_3_ − 0.0019 × B_2_O_3_ × NaF − 0.0016 × B_2_O_3_ × KF + 0.0015 × CaCO_3_ × Na_2_CO_3_	0.99	0.99	0.97	66.66
Tg Onset	5.11 × B_2_O_3_ + 6.12 × MgO + 7.69 × CaCO_3_ + 2.41 × Na_2_CO_3_ + 2.98 × NaF + 1.25 × KF	0.84	0.80	0.74	17.83
Tg Inflection	4.17 × B_2_O_3_ + 3.06 × MgO + 0.99 × CaCO_3_ − 2.96 × Na_2_CO_3_ + 3.87 × NaF + 2.38 × KF + 0.092 × B_2_O_3_ × MgO + 0.16 × B_2_O_3_ × CaCO_3_ + 0.14 × B_2_O_3_ × Na_2_CO_3_ − 0.067 × MgO × Na_2_CO_3_ + 0.066 × MgO × KF − 0.10 × CaCO_3_ × Na_2_CO_3_ + 0.10× CaCO_3_ × NaF	0.99	0.98	0.83	35.24
Tg Final	4.35 × B_2_O_3_ + 9.32 × MgO + 1.83 × CaCO_3_ − 1.89 × Na_2_CO_3_ + 3.75 × NaF + 2.11 × KF + 0.14 × B_2_O_3_ × CaCO_3_ + 0.11 × B_2_O_3_ × Na_2_CO_3_ − 0.087 × MgO × CaCO_3_ − 0.14 × MgO × Na_2_CO_3_ − 0.090 × MgO × NaF − 0.11 × CaCO_3_ × Na_2_CO_3_ + 0.10 × CaCO_3_ × NaF	0.99	0.98	0.88	33.42
1 h Mass Loss	(100 × e*^y^*)/(1 + e*^y^*) where y = 0.10 × B_2_O_3_ + 0.17 × MgO + 0.88 × CaCO_3_ + 0.010 × Na_2_CO_3_ − 0.061 × NaF − 0.036 × KF − 0.0059 × B_2_O_3_ × MgO − 0.021 × B_2_O_3_ × CaCO_3_ − 0.0032 × CaCO_3_ × Na_2_CO_3_	0.94	0.92	0.86	18.12
12 h Mass Loss	(100 × e*^y^*)/(1 + e*^y^*) where y = 0.045 × B_2_O_3_ − 0.026 × MgO − 0.055 × CaCO_3_ + 0.046 × Na_2_CO_3_ + 0.0024 × NaF − 0.00077 × KF	0.76	0.70	0.59	12.32
24 h Mass Loss	(100 × e*^y^*)/(1 + e*^y^*) where y = 0.045 × B_2_O_3_ − 0.027 × MgO + −0.054 × CaCO_3_ + 0.043 × Na_2_CO_3_ + 0.0022 × NaF + 0.0010 × KF	0.74	0.67	0.55	11.51
1 h Fluoride Release	(2000 × e*^y^*)/(1 + e*^y^*) where y = −0.057 × B_2_O_3_ − 0.015 × MgO − 0.045 × CaO+ 0.18 × Na_2_O + 0.031 × KF + 0.14 × NaF − 0.0045 × CaO × NaF + 0.0039 × Na_2_O × KF.	0.85	0.79	0.70	11.68
12 h Fluoride Release	(2000 × e*^y^*)/(1 + e*^y^*) where y = −0.053 × B_2_O_3_ − 0.027 × MgO − 0.040 × CaCO_3_ + 0.080 × Na_2_CO_3_ -0.20 × NaF + 0.036 × KF − 0.0019 × B_2_O_3_ × Na_2_CO_3_ + 0.0050× B_2_O_3_ × NaF + 0.0031 × MgO × Na_2_CO_3_ + 0.0029 × Na_2_CO_3_ × NaF − 0.0026 × NaF × KF	0.95	0.90	0.76	18.31
24 h Fluoride Release	(2000 × e*^y^*)/(1 + e*^y^*) where y = −0.059 × B_2_O_3_ − 0.0029 × MgO − 0.0384 × CaCO_3_ + 0.014 × Na_2_CO_3_ − 0.14 × NaF + 0.080 × KF + 0.0035 × B_2_O_3_ × NaF − 0.0016 × MgO × KF − 0.0020 × CaCO_3_ × KF + 0.0021 × Na_2_CO_3_ × NaF	0.98	0.96	0.84	29.5

**Table 5:** The mass loss and fluoride release values following incubation of glass powders in TRIS-buffered saline, compositions without reported values yielded insufficient materials for processing and analysis.

	Mass Loss (%)			Fluoride Release (ppm)		
	1 h	12 h	24 h	1 h	12 h	24 h
1	-	-	-	-	-	-
2	99.8	98.4	98.4	168.4	25.2	74.6
3	53.5	79.5	79.5	360.6	318.6	152.5
4	93.7	91.9	91.9	1918.1	946.9	582.5
5	82.8	79.1	79.1	714.6	459.1	191.0
6	-	-	-	-	-	-
7	98.6	93.2	93.2	670.4	463.5	201.0
8	61.9	73.0	73.0	500	448.0	165.0
9	-	-	-	-	-	-
10	81.6	82.3	82.3	1342.9	177.7	483.0
11	97.8	92.4	92.4	1725.7	394.7	1064.5
12	96.0	95.2	95.2	1216.8	273.9	766.5
13	-	-	-	-	-	-
14	47.9	61.1	61.1	39.6	46.7	49.2
15	55.1	81.9	81.9	674.8	98.2	202.0
16	72.5	73.5	62.7	112	111.5	94.7
17	-	-	-	-	-	-
18	56.9	60.8	64.7	71.9	81.1	79.5
19	96.8	99.2	99.2	1915.9	418.9	854.5
20	-	-	-	-	-	-
21	65.5	59.7	64.3	82.9	66.0	71.5
22	99.7	95.9	95.9	119.4	113.7	223.0
23	98.0	99.4	99.4	149.2	144.7	329.0
24	92.6	96.0	96.0	441.2	414.5	808.0
25	40.5	55.0	59.3	16.9	21.3	21.2
26	67.8	85.9	85.9	127.5	115.2	187.0
27	92.1	91.9	93.0	137.9	134.5	117.9
28	64.4	93.8	93.8	50.5	64.5	64.7
29	76.3	97.2	97.2	14.4	16.6	16.7
30	48.3	62.8	62.3	41.6	50.7	52.1
31	66.9	67.8	69.6	110.4	103.4	95.2

## Data Availability

The data presented in this study is the property of IR Scientific and is available on request from the corresponding author.

## References

[1] Mouriño V., Cattalini J.P., Boccaccini A.R. (2012). Metallic ions as therapeutic agents in tissue engineering scaffolds: An overview of their biological applications and strategies for new developments. J. R. Soc. Interface.

[2] Habibovic P., Barralet J.E. (2011). Bioinorganics and biomaterials: Bone repair. Acta Biomater..

[3] Fernandes J.S., Martins M., Neves N.M., Fernandes M.H.V., Reis R.L., Pires R.A. (2016). Intrinsic antibacterial borosilicate glasses for bone tissue engineering applications. ACS Biomater. Sci. Eng..

[4] Day R.M. (2005). Bioactive glass stimulates the secretion of angiogenic growth factors and angiogenesis in vitro. Tissue Eng..

[5] Fernandes J.S., Gentile P., Crawford A., Pires R.A., Hatton P.V., Reis R.L. (2017). Substituted borosilicate glasses with improved osteogenic capacity for bone tissue engineering. Tissue Eng. Part A.

[6] Schmitz S., Widholz B., Essers C., Becker M., Tulyaganov D., Moghaddam A., de Juan I.G., Westhauser F. (2020). Superior biocompatibility and comparable osteoinductive properties: Sodium-reduced fluoride-containing bioactive glass belonging to the CaO–MgO–SiO_2_ system as a promising alternative to 45S5 bioactive glass. Bioact. Mater..

[7] Jones J.R. (2013). Review of bioactive glass: From Hench to hybrids. Acta Biomater..

[8] Wray P. (2011). Cotton candy that heals? Borate glass nanofibers look promising. Am. Ceram. Soc. Bull..

[9] Castiglioni S., Cazzaniga A., Albisetti W., Maier J.A.M. (2013). Magnesium and osteoporosis: Current state of knowledge and future research directions. Nutrients.

[10] Rondanelli M., Faliva M.A., Tartara A., Gasparri C., Perna S., Infantino V., Riva A., Petrangolini G., Peroni G. (2021). An update on magnesium and bone health. Biometals.

[11] Wopenka B., Pasteris J.D. (2005). A mineralogical perspective on the apatite in bone. Mater. Sci. Eng. C.

[12] Bigi A., Falini G., Foresti E., Gazzano M., Ripmonti A., Roveri N. (1996). Rietveld structure refinements of calcium hydroxylapatite containing magnesium. Acta Crystallogr. Sect. B Struct. Sci..

[13] Abdallah M.-N., Eimar H., Bassett D.C., Schnabel M., Ciobanu O., Nelea V., McKee M.D., Cerruti M., Tamimi F. (2016). Diagenesis-inspired reaction of magnesium ions with surface enamel mineral modifies properties of human teeth. Acta Biomater..

[14] Abdallah M.N. (2012). Surface Reactivity of Tooth Enamel with Dyes Oxidizing Agents and Magnesium Ions and Its Effect on Tooth Color. Master’s Thesis.

[15] Shaikh K., Pereira R., Gillam D.G., Phad S. (2018). Comparative evaluation of desensitizing dentifrices containing BioMin^®^, Novamin^®^ and fluoride on dentinal tubule occlusion before and after a citric acid challenge—A scanning electron microscope in-vitro study. J. Odontol..

[16] Reddy G.V., Surakanti J.R., Vemisetty H., Doranala S., Hanumanpally J.R., Malgikar S. (2019). Comparative assessment of effectiveness of Biomin, NovaMin, herbal, and potassium nitrate desensitizing agents in the treatment of hypersensitive teeth: A clinical study. J. NTR Univ. Health Sci..

[17] Tirapelli C., Panzeri H., Soares R.G., Peitl O., Zanotto E.D. (2010). A novel bioactive glass-ceramic for treating dentin hypersensitivity. Braz. Oral Res..

[18] De Caluwe T., Vercruysse C.W.J., Declercq H.A., Schaubroeck D., Verbeeck R.M.H., Martens L.C. (2016). Bioactivity and biocompatibility of two fluoride containing bioactive glasses for dental applications. Dent. Mater..

[19] MacDonald K., Boudreau E., Thomas G.V., Badrock T.C., Davies L.J., Lloyd M.J., Spradbery P.S., Turner-Cahill S., Boyd D. (2021). In vitro evaluation of Sensi-IP^®^: A soluble and mineralizing sensitivity solution. Heliyon.

[20] Balasubramanian P., Büttner T., Pacheco V.M., Boccaccini A.R. (2018). Boron-containing bioactive glasses in bone and soft tissue engineering. J. Eur. Ceram. Soc..

[21] Liang W., Rüssel C., Day D.E., Völksch G. (2006). Bioactive comparison of a borate, phosphate and silicate glass. J. Mater. Res..

[22] Goetschius K.L., Beuerlein M.A., Bischoff C.M., Brow R.K. (2018). Dissolution behavior of ternary alkali–alkaline earth-borate glasses in water. J. Non-Cryst. Solids.

[23] Wright A.C., Dalba G. (2010). Borate versus silicate glasses: Why are they so different?. Phys. Chem. Glasses Eur. J. Glass Sci. Technol. B.

[24] Wright A.C. (2015). My borate life: An enigmatic journey. Int. J. Appl. Glass Sci..

[25] Hill R. (1996). An alternative view of the degradation of bioglass. J. Mater. Sci. Lett..

[26] Manchester R.A., Todorova T.Z., Werner-Zwanziger U., Boyd D. (2021). Mixture designs to investigate the role of alkali and alkaline earth cations on composition–structure–property relationships in ternary borate glass networks. J. Non-Cryst. Solids.

[27] MacDonald K., Hanson M.A., Boyd D. (2016). Modulation of strontium release from a tertiary borate glass through substitution of alkali for alkali earth oxide. J. Non-Cryst. Solids.

[28] Yiannopoulos Y.D., Chryssikos G.D., Kamitsos E.I. (2001). Structure and properties of alkaline earth borate glasses. Phys. Chem. Glasses.

[29] Kamitsos E.I., Chryssikos G.D., Karakassides M.A. (1987). Vibrational spectra of magnesium-sodium-borate glasses. 1. Far-infrared investigation of the cation-site interactions. J. Phys. Chem..

[30] Doweidar H., El-Egili K., Ramadan R., Khalil E. (2019). Structural species in mixed-fluoride PbF_2_–CdF_2_–B_2_O_3_ borate glasses; FTIR investigation. Vib. Spectrosc..

[31] El-Egili K., Doweidar H., Ramadan R., Altawaf A. (2016). Role of F− ions in the structure and properties of BaF_2_B_2_O_3_ glasses. J. Non-Cryst. Solids.

[32] Doweidar H., El-Egili K., Ramadan R., Khalil E. (2018). Structural studies and properties of CdF_2_–B2O_3_ glasses. J. Non-Cryst. Solids.

[33] Doweidar H., El-Egili K., Altawaf A. (2017). Structural units and properties of BaF_2_–PbF_2_–B_2_O_3_ glasses. J. Non-Cryst. Solids.

[34] Doweidar H., El-Damrawi G., Abdelghany M. (2012). Structure and properties of CaF_2_–B2O_3_ glasses. J. Mater. Sci..

[35] ElBatal F.H., Ouis M.A., ElBatal H.A. (2016). Comparative studies on the bioactivity of some borate glasses and glass–ceramics from the two systems: Na_2_O–CaO–B_2_O_3_ and NaF–CaF_2_–B_2_O_3_. Ceram. Int..

[36] Ouis M.A., Abdelghany A.M., Elbatal H.A. (2012). Corrosion mechanism and bioactivity of borate glasses analogue to Hench’s bioglass. Process. Appl. Ceram..

[37] Mauro J.C., Philip C.S., Vaughn D.J., Pambianchi M.S. (2014). Glass science in the United States: Current status and future directions. Int. J. Appl. Glass Sci..

[38] Han T., Stone-Weiss N., Huang J., Goel A., Kumar A. (2020). Machine learning as a tool to design glasses with controlled dissolution for healthcare applications. Acta Biomater..

[39] Krishnan N.A., Mangalathu S., Smedskjaer M.M., Tandia A., Burton H., Bauchy M. (2018). Predicting the dissolution kinetics of silicate glasses using machine learning. J. Non-Cryst. Solids.

[40] Ahmmad S.K., Jabeen N., Ahmed S.T.U., Hussainy S.F., Ahmed B. (2021). Density of fluoride glasses through artificial intelligence techniques. Ceram. Int..

[41] Ahmmad S.K., Jabeen N., Ahmed S.T.U., Ahmed S.A., Rahman S. (2021). Artificial intelligence density model for oxide glasses. Ceram. Int..

[42] Wright A.C. (2010). Borate structures: Crystalline and vitreous. Phys. Chem. Glasses Eur. J. Glass Sci. Technol..

[43] Aguiar P.M., Kroeker S. (2007). Boron speciation and non-bridging oxygens in high-alkali borate glasses. J. Non-Cryst. Solids.

[44] Sokolov I.A., Naraev V.N., Nosakin A.N., Pronkin A.A. (2000). Influence of MeF_2_ (Me = Mg, Ca, Sr, and Ba) on the electrical properties of glasses in the MeF_2_-Na_2_B_4_O_7_ system. Glass Phys. Chem..

[45] Abdelghany A.M., Kamal H. (2014). Spectroscopic investigation of synergetic bioactivity behavior of some ternary borate glasses containing fluoride anions. Ceram. Int..

[46] Chowdari B.V.R., Rong Z. (1995). Study of the fluorinated lithium borate glasses. Solid State Ion..

[47] Shaharyar Y., Wein E., Kim J.-J., Youngman R.E., Muñoz F., Kim H.-W., Tilocca A., Goel A. (2015). Structure-solubility relationships in fluoride-containing phosphate based bioactive glasses. J. Mater. Chem. B.

[48] Lusvardi G., Malavasi G., Cortada M., Menabue L., Menziani M.C., Pedone A., Segre U. (2008). Elucidation of the structural role of fluorine in potentially bioactive glasses by experimental and computational investigation. J. Phys. Chem. B.

[49] Brauer D.S., Karpukhina N., Law R.V., Hill R.G. (2009). Structure of fluoride-containing bioactive glasses. J. Mater. Chem..

[50] Gaafar M.S., Marzouk S.Y., Zayed H.A., Soliman L.I., El-Deen A.H.S. (2013). Structural studies and mechanical properties of some borate glasses doped with different alkali and cobalt oxides. Curr. Appl. Phys..

[51] Lower N.P., McRae J.L., Feller H.A., Betzen A.R., Kapoor S., Affatigato M., Feller S.A. (2001). Physical properties of alkaline-earth and alkali borate glasses prepared over an extended range of compositions. J. Non-Cryst. Solids.

[52] Doweidar H., El-Damrawi G., Mansour E., Fetouh R.E. (2012). Structural role of MgO and PbO in MgO–PbO–B_2_O_3_ glasses as revealed by FTIR; A new approach. J. Non-Cryst. Solids.

[53] Deng B. (2020). Machine learning on density and elastic property of oxide glasses driven by large dataset. J. Non-Cryst. Solids.

[54] Kamitsos E.I. (1989). Modifying role of alkali-metal cations in borate glass networks. J. Phys. Chem..

[55] Varshneya A.K. (2013). Fundamentals of Inorganic Glasses.

[56] Zhong J., Bray P.J. (1988). Change in boron coordination in alkali borate glasses, and mixed alkali effects, as elucidated by NMR. JNCS.

[57] Bachar A., Mercier C., Tricoteaux A., Hampshire S., Leriche A., Follet C. (2013). Effect of nitrogen and fluorine on mechanical properties and bioactivity in two series of bioactive glasses. J. Mech. Behav. Biomed..

[58] Huang W., Day D.E., Kittiratanapiboon K., Rahaman M.N. (2006). Kinetics and mechanisms of the conversion of silicate (45S5), borate, and borosilicate glasses to hydroxyapatite in dilute phosphate solutions. J. Mater. Sci. Mater. Med..

[59] Brauer D.S., Karpukhina N., O’Donnell M.D., Law R.V., Hill R.G. (2010). Fluoride-containing bioactive glasses: Effect of glass design and structure on degradation, pH and apatite formation in simulated body fluid. Acta Biomater..

[60] Brauer D.S., Mneimne M., Hill R.G. (2011). Fluoride-containing bioactive glasses: Fluoride loss during melting and ion release in tris buffer solution. J. Non-Cryst. Solids.

[61] Shah F.A. (2016). Fluoride-containing bioactive glasses: Glass design, structure, bioactivity, cellular interactions, and recent developments. Mater. Sci. Eng. C.

